# Exploring the impact of perceived risk and trust on tourist acceptance intentions in the post-COVID-19 era: A case study of Hainan residents

**DOI:** 10.3389/fpsyg.2022.934425

**Published:** 2022-10-26

**Authors:** Hongxia Zhou, Johan Afendi Bin Ibrahim, Ahmad Edwin Bin Mohamed

**Affiliations:** ^1^College of Tourism, Hainan University, Haikou, China; ^2^School of Tourism, Hospitality and Event Management, Universiti Utara Malaysia, Sintok, Malaysia

**Keywords:** tourism of COVID-19, trust, perceived risk, acceptance intention of Hainan resident, theory of reasoned action

## Abstract

Hainan, is the only free trade port that also exudes quintessence of the culture of China. Tourism is one of Hainan's most lucrative industries. On the one hand, the regional economy is flourishing and on the other hand, the economy is facing unprecedented impacts from the COVID-19 pandemic. In response to the affected global market environment, this study investigates Hainan residents' acceptance intentions, or tolerance, of tourists. Here, based on the theory of reasoned action, which includes “subjective norm” combined with “trust,” “perceived risk,” and “perceived value.” Using “resident attitude” as a mediator, theoretical research frameworks were developed. A total of 447 valid responses were collected using online and paper-copy questionnaires distributed to Hainan residents from 15 July 2021 to 10 November 2021. The data from the questionnaires were used for three analyses namely, descriptive statistical analysis, measurement model verification, and structural equation modeling analysis. Findings show a positive effect of trust on residents' attitudes in Hainan; perceived value and subjective norm showed a positive effect on resident acceptance intentions for tourism; the perceived risk of residents showed a more negative effect on attitudes toward tourists, but the influence was small. Finally, through the results of the study, theoretical and practical implications in a post-pandemic era are discussed.

## Introduction

The devastation and crisis caused by the COVID-19 global pandemic have resulted in major socioeconomic upheaval (Prime et al., 2020). The multiplicity of pandemic pathogens and their recurrence and rebound in multiple regions is unprecedented. To date, the World Health Organization is advising countries and individuals to take steps in protecting their health and preventing the spread of the pandemic (WTO, 2021). As a result of COVID-19, the world's tourism industry is facing a disaster beyond imagination as tourism is vulnerable to natural disasters, pandemics, conflicts, terrorism, and economic crises. For example, health measures and communication restrictions such as embargoes, travel bans, quarantine, and social distancing have halted business in tourism-related industries. COVID-19 is causing a long-term structural change in the tourism industry and challenging existing economic and tourism systems. However, tourism has shown resiliency in rebounding from major economic, political, and health crises in the past (Sigala, 2020). Even now, with the recurrence of the pandemic in individual regions, people's demand for tourism is still rising and the market demand for cross-regional and overseas tourism is substantial. Therefore, the management of pandemic precautions in tourism is an important and long-term social issue facing the world today.

According to a recent study released by the World Travel and Tourism Council (WTTC) on tourism global averages, Chinese tourism recovery is significantly ahead of other key markets in Europe and the United States. With several government initiatives underway, the tourism market is predicted to be extremely optimistic for 2022 (WTTC, 2021). According to official data from China's Hainan Tourism and Culture, Radio, Film, and Sports, Hainan Province received 81.43 million domestic and foreign tourists in 2021 which is up an average of 25.5% per year since 2019 and, recovering to 97.5%. Total tourism revenue was 138.434 billion RMB in 2021, up an average of 58.6 and 30.9% since 2019. Hainan has one of the best tourism recovery rates in China (QiuShi, 2022). Enthusiasm and demand for cross-regional travel are still evident. Hainan is a free trade port with the quintessence of Chinese cultural characteristics and a destination for tourists. The region is currently building a pilot free trade zone and the tourism industry is an economic pillar of Hainan (SCMP, 2022). However, with the recurrence and uncertainty of the pandemic, residents of tourist destinations have heightened perceived risk and trust regarding safety associated with sightseeing tourists. Residents' wariness of foreign tourists is increasing, especially toward tourists from high-risk areas located outside China. Nonetheless, tourism companies are surviving despite the turmoil of the pandemic and are trying to seize the potential of tourism opportunities. For regions and countries where tourism is an important economic pillar, a safe living environment is essential for residents of tourist destinations. As an exemplary tourist destination, Hainan's community deserves a more in-depth exploration of the attitudes, perceived risks, and trust of residents toward tourists.

Although the body of research on COVID-19 and tourism is growing, past research has mainly focused on the impact on the tourism industry (Baum and Hai, 2020; Lew et al., 2020; Ranasinghe et al., 2020; Sigala, 2020). Several studies have focused on the destination-image recovery processes (Castillo-Villar, 2020; Chemli et al., 2020; Rasoolimanesh et al., 2021) and estimating impacts and changes in visitor behavior (Davahli et al., 2020; McGinlay et al., 2020; Wen et al., 2020). However, because tourism is the main source of income for the long-term livelihood of residents in tourism destinations, empirical studies should consider the resident's acceptance intention, or tolerance, for tourists, especially under the norm of recurrent pandemics. This study employs the theory of reasoned action (TRA) while using the impact of residents' perceptions (e.g., perceived risk and trust) to develop a theoretical structural model of tourism in the context of COVID-19. Perceived value is considered a construct of perceived behavior control in TRA. The application of TRA to residents' acceptance intention of tourism is critical to comprehensively augment a structural framework of tourism tolerance.

Therefore, this study takes Hainan residents' intention to accept tourists as the main axis, and uses trust, perceived risk, perceived value, and subjective norm as independent variables. Attitude is considered a mediating variable to explore the mechanism of factors influencing Hainan residents' intention to accept tourist visitors. Based on domestic and international literature around tourism, this study designed questions aligning it with the current research context, used questionnaires as a means of collecting data, and conducted data analysis using statistical tools. This study's results serve to highlight practical implications and provide managerial recommendations for tourism practitioners and local tourism-related government departments.

## Theoretical model construction and research hypotheses

### Theory of reasoned action

The Theory of Reasoned Action is a foundational and highly influential theory in social psychology used to study cognitive behavior and was originally developed by Fishbein (1967) and Ajzen and Fishbein (1969). The four main factors of the TRA model include attitude, subjective norm, actual behavior, and behavioral intention. The theory asserts that behavior precedes intention; an individual's intention is determined by subjective normative attitudes (Fishbein et al., 1980). Individual behavioral attitudes and subjective norms lead to behavioral intentions, and individual behavioral intentions directly influence actual behavior (Ajzen and Fishbein, 1975). This suggests that human rational behavior satisfies both individual's wishes and the expectations of others (Dan and Chieh, 2008). Attitude refers to the perception and evaluation of performing a specific behavior and includes consideration of the subsequent outcome (Verma and Sinha, 2018). Subjective norms are the views and opinions held about social pressures when engaging in specific behavior and are easier or more difficult to express if others hold favorable or unfavorable opinions, respectively (Procter et al., 2019). Ajzen and Fishbein (1975) originally defined intention as the expectation of one's behavior in a given context, operationalized as the likelihood of an individual performing an act. The intention is influenced by two variables - attitudes and subjective norms. In the context of this study, attitudes refer to Hainan residents' positive or negative perceptions of tourists after COVID-19. Subjective norm refers to how residents are influenced by the perceptions of friends, neighbors, co-workers, family members, and community residents about tourists. The intention is the extent to which residents accept or tolerate tourists.

Several studies have explored human behavior based on TRA. Past research based on TRA focused on issues such as gambling (Procter et al., 2019), purchasing, and user behaviors. For instance, Chen et al. (2020) and Loan and Quyen (2020) investigated consumer attitudes toward purchasing social insurance, using smart home devices, and purchasing environmentally sustainable goods. The theory is also widely used in health and medical-related studies to explain increases in healthy behaviors correlating with a decrease in habitual behaviors (Sheeran and Conner, 2019). For example, a study was conducted about college students and their participation in health-related extracurricular activities, where attitudes, subjective norms, and cultural exploration have significant positive effects on college students' behavioral intentions to participate in college activities (Harb et al., 2021). In the field of tourism, Song et al. (2021) examined the behavior relating to Chinese golfers' intention to revisit, which was influenced by the golfers' perceptions of staff attitudes, destination uniqueness, and place attachment.

Results from several studies suggest a positive correlation between trust and attitude. For example, Sadiq et al. (2021) studied online travel purchases and concluded trust strongly influences consumer attitudes toward online purchases. In addition, Ng (2020) proposed trust in colleagues is a catalyst for influencing employees' willingness to share knowledge with others. An increase in trust with colleagues positively impacts attitudes toward knowledge sharing. Informed by past studies utilizing TRA, the results of this study are likely to find that increased trust of Hainan residents toward tourists simultaneously increases positive attitudes. The increase in trust of Hainan residents is likely to increase the influence of tourists' attitudes. Therefore, this study proposes hypothesis 1.

H1: The trust of residents in Hainan toward tourists will have a significantly positive effect on attitudes toward tourists.

#### Trust

Trust is defined as the willingness of one party to believe or rely on the other party's attitude or willingness to have a mutually beneficial human interaction (Moorman et al., 1993; McKnight and Chervany, 2001; Kim and Tadisina, 2007). Trust is also defined as the perceived feeling of reliability and goodwill of one party toward the other party (Gefen and Straub, 2004). McCarter and Northcraft (2007) found that trust is a psychological situation in which one party is willing to believe in another party in anticipation of cooperation. Social scientists are increasingly interested in the concept of trust as a response to incomplete knowledge that leads to uncertainty (Giddens et al., 1991). Lewis and Weigert (1985) stated, “trust begins where prediction ends,” and tourism, like all areas of life, is fraught with uncertainty. One focus of this study is on trust interactions between travelers and residents. Given that COVID-19 spreads from person to person, all people in the same spatial proximity should have baseline trust to protect each other (Dedeoglu and Bogan, 2021; Ukpabi et al., 2021; Quintal et al., 2022). Shin et al. (2022) indicate future travel intentions would be determined by an individual's level of trust in the COVID-19 measures at their destination. Here, this paper proposes Hainan residents' trust in tourism flourishes when tourists choose to do the right thing for local residents. In return, local Hainan residents are willing to establish trust and create mutually beneficial interactions.

Trust includes social trust, government trust, social media trust, and online perception trust. Trust in these sectors has increased to an unprecedented level. For instance, trust leads to higher purchase intentions in online shopping environments (Jones and Kim, 2010). Additionally, Kim et al. (2012) and Pan et al. (2012) verified online shopping intentions are influenced by consumer trust, and trust also influences consumers' perceptions of online shopping. Trust is critical in a rapidly evolving event like COVID-19, which is characterized by scientific uncertainty (Balog-Way and McComas, 2020). Travel-related businesses are now compelled to find strategies to retain customers. One basic strategy is to build customer trust and loyalty. (Laparojkit and Suttipun, 2021). During times of uncertainty, trust is a key factor in sustaining society and underpins people's attitudes and behaviors (Paul et al., 2021).

#### Perceived risk

Bauer (1960) argued most consumer behaviors may be risky behavior and consumers perceive risk because they are unable to predict outcomes from their purchases and the approximate probability of various outcomes. Perceived risk in tourism is defined as an individual's perception of “behaviors that may influence travel decisions if hazards are perceived to be beyond acceptable levels” (Chew and Jahari, 2014). Risks may include physical, psychological, financial, and health risks from injuries, accidents, terrorism, natural disasters, political instability, and pandemics. Some outcomes of purchases may be negative and once perceived as potentially negative by consumers, they are then typically perceived as a risk. In the tourism industry, risk is considered a major concern for international travelers (Kozak et al., 2007). Due to tourists inherently seeking security, travel decisions made in situations of uncertain risk can be heavily influenced by safety and security concerns (Beirman, 2002). In addition, the experiential and intangible nature of tourism often leads to higher levels of non-systematic risk perceived by tourists (Fuchs, 2013). The perceived risk in this study is Hainan residents risking their health due to coronavirus disease potentially being transmitted by tourists. Due to the risk COVID-19 poses, tourism increases the risk for Hainan residents to unknown levels. Therefore, perceived risk, as it relates to the transmission of coronavirus, is defined as the uncertainty of risk residents of Hainan face.

Since the 1990's, researchers have studied various pandemics' impacts on tourism decisions and tourist behavior (Li et al., 2020; Choe et al., 2021; Sánchez-Cañizares et al., 2021). In particular, the economic impact of pandemics on tourism and tourism intentions have been widely discussed. For instance, severe diseases such as SARS, avian influenza, and the Middle East respiratory syndrome have severely affected the tourism industry (Floyd et al., 2004; Lee et al., 2012). The global outbreak and prevalence of COVID-19 in just 3 years have led to an increase in travel-related empirical studies. Indeed, objective risks may only have an impact on travelers' behavior when they are perceived. Similarly, residents face several risks when receiving foreign tourists into their country, like, compromised privacy, security problems in tourist destinations, and information about health protocols.

Numerous studies have been conducted relating to the relationship between perceived risk and attitudes. Andrews et al. (2014) investigated Australian public perception of personally controlled electronic health records and results show that perceived risk negatively affects attitudes. Another study about online consumer purchase behavior suggested that perceived risk has a significant role on attitude (Sadiq et al., 2021). Notably, Bae and Chang (2021) examined the effect of perceived risk from COVID-19 on willingness to travel “non-contact” during the first wave of the pandemic in Korea in March 2020. Results suggest emotional risk perception has a significantly positive effect on attitudes toward non-contact travel. Based on these literature, this study proposes attitudes defined as the positive or negative perceptions of Hainan residents toward tourists after COVID-19. The subjective norm is the magnitude of influence that perceptions of friends, neighbors, co-workers, family, and community residents on visitors have on Hainan residents. The intention is the degree of acceptance toward tourists by residents, and when the perceived risk of residents to tourists increases, attitude toward tourists is affected. Additionally, the higher the perceived risk, the more negative the attitude is. Therefore, this study proposes hypothesis 2.

H2: Attitudes toward tourists by residents in Hainan have a significantly negative effect on the perceived risk of tourists.

#### Perceived value

Perceived value is defined as the consumer's overall assessment of the utility which is based on perceptions of what is received and given (Zeithaml, 1988). Consumer-perceived value is the consumer's perceived performance-to-price ratio, and consumers' price perception has a strong influence on the customer's perceived value (Varki and Colgate, 2001). Perceived value is measured by assessing the range of consumer experiences (Sweeney and Soutar, 2001) and measuring the difference between actual costs and perceived benefits (Gallarza and Saura, 2006). In this study, perceived value is the perception of Hainan residents after COVID-19 reflecting the actual local needs and tourism as a positive local economic effect, i.e., visitors are welcome. Moreover, the perceived value is defined as the perception of residents of Hainan that tourists bring direct and positive economic benefits.

Many empirical studies on tourism focus on perceived value. Young tourists' perception values of nature-based tourism experiences and impacts on trip outcomes include overall satisfaction, word of mouth, and intent to revisit (Caber et al., 2020). Perceived value, destination image, and tourist satisfaction in war tourism predict the indirect and direct effects of quality tourism experiences on behavioral intentions (Ghorbanzadeh et al., 2021). The perceived value of upscaling experiences on tourists affects their attitudes and intentions to travel responsibly, and how their attitudes moderate intentions (Um and Yoon, 2021). Tourism motivation enhances the quality of experience and perceived value (Lu et al., 2021). The value of tourism is enhanced by improving positioning strategies and the promotion of tourism niches (Jamal et al., 2011). One study concluded that among trust and habit, perceived value is an influencing factor explaining the intention to travel via air. Um and Yoon (2021) concluded perceived condition value of tourism upscaling on the intention to conserve, indicating the condition value of tourism areas. In addition, Caber et al. (2020) estimated young visitors' perceived value as an important determinant of overall satisfaction and behavioral intentions (i.e., word-of-mouth and revisit intentions). Several studies show individuals' performance and effort expectations indirectly influence their adopted intentions through perceived value.

According to the above literature, this study builds on the past exploration of the perceived value as related to Hainan residents' perception of tourists under the influence of the COVID-19 pandemic. Here, this study infers that the perceived value of local residents toward tourists has a positive impact on acceptance intention, and the more positive the perceived value, the higher the tolerance to tourists. This study proposes hypothesis 3.

H3: Residents in Hainan have a significant positive effect on the perceived value of tourists on their acceptance intention toward tourists.

#### Attitude, subjective norms, and resident acceptance intention

Numerous studies have investigated the correlation and positive effects between attitudes and intention to adopt. Krajaechun and Praditbatuga (2019) articulated their results using Pearson's correlation coefficient, showing the acceptability of non-life insurance conditions and subjective normative attitudes toward the product were significantly associated with the intention to purchase non-life insurance. Nomi and Sabbir (2020) examined individual-level factors to explain changes in intention to purchase life insurance, empirically testing TRA as valuable. Um and Yoon (2021) argued that attitudes influence the intention to protect and participate in responsible tourism. Nomi and Sabbir (2020) found that attitudes and subjective norms have the greatest impact on purchase intentions.

The positive correlation and effects between subjective norms and adoption intentions are frequently studied. For example, Polat et al. (2021) expanded trust and social norms in airline services, directly and indirectly, affecting air travel intentions by reducing perceived risks. Firouzbakht et al. (2021) found moderating health beliefs and subjective norms (indirect beta = 0.35) exert a greater indirect effect on behavioral intentions and a higher intention coefficient (β = 0.626) was observed for subjective norms. This study draws from previous studies to formulate hypotheses 4 and 5 to examine the acceptance of tourists by residents of Hainan impacted by the COVID-19 pandemic. It can be inferred that the attitude of residents toward tourists has a positive effect on their acceptance, and the more positive the attitude, the higher the acceptance of tourists. For the same reason, residents' perceptions of tourists are influenced by their friends and relatives, and the higher the degree of subjective norm influence, the higher the acceptance of domestic and overseas tourists. Therefore, hypotheses 4 and 5 are proposed in this study.

H4: The attitude of residents of Hainan toward tourists has a positive and significant effect on the intention to accept tourists.
H5: There is a positive and significant effect of Hainan residents' subjective norms toward tourists on the intention to accept tourists.

#### Trust, perceived risk, attitude, and resident acceptance intention

Research on the presence and effects of attitudes as a mediating variable are numerous. In Harb et al. (2021), attitudes were found to mediate the effects of cultural exploration and subjective norms on students' behavioral intentions. Also, Um and Yoon (2021) verified the mediating role of attitudes in the relationship between tourists' perceived experience value and intention to travel responsibly. Additionally, many studies investigated attitudes as a mediating effect between trust and intention to travel. As stated by Kasri and Ramli (2019), Muslim donations in Indonesia found that attitudes had a mediating effect between trust and intention to donate. According to Shin et al. (2022), the COVID-19 pandemic forced travel-related agencies to develop effective strategies to attract travelers and developed an integrated framework to explain the effects of the pandemic period and post-pandemic travel facilitation, constraints, and attitudinal factors on travel decisions. The results showed the specific factors determining travel decisions (travel intentions and frequency) during and after a pandemic. The results reflected that attitude had a mediating effect between trust and intention to travel.

Attitudes also have a mediating effect between perceived risk and intention to accept. Hung et al. (2006) explored the e-Government service and found attitudes toward utilization of the service have a mediating effect between perceived risk and intention to accept. Further, Andrews et al. (2014) declared that Australian residents' attitudes toward personally controlled electronic health records have a mediating effect between perceived risk and intention to accept. Therefore, according to the above literature, residents' attitudes toward foreign tourists should have a mediating effect between their trust and acceptance intention. Simultaneously, residents' attitudes toward foreign tourists should have a mediating effect between their perceived risk and intention to accept. Accordingly, hypotheses 6 and 7 are proposed in this study.

H6: Residents' attitudes toward tourists have a mediating effect on trust and acceptance intention.
H7: A mediating effect exists in Hainan residents' attitude toward tourists between perceived risk and acceptance intention of tourists.

**Figure 1:**
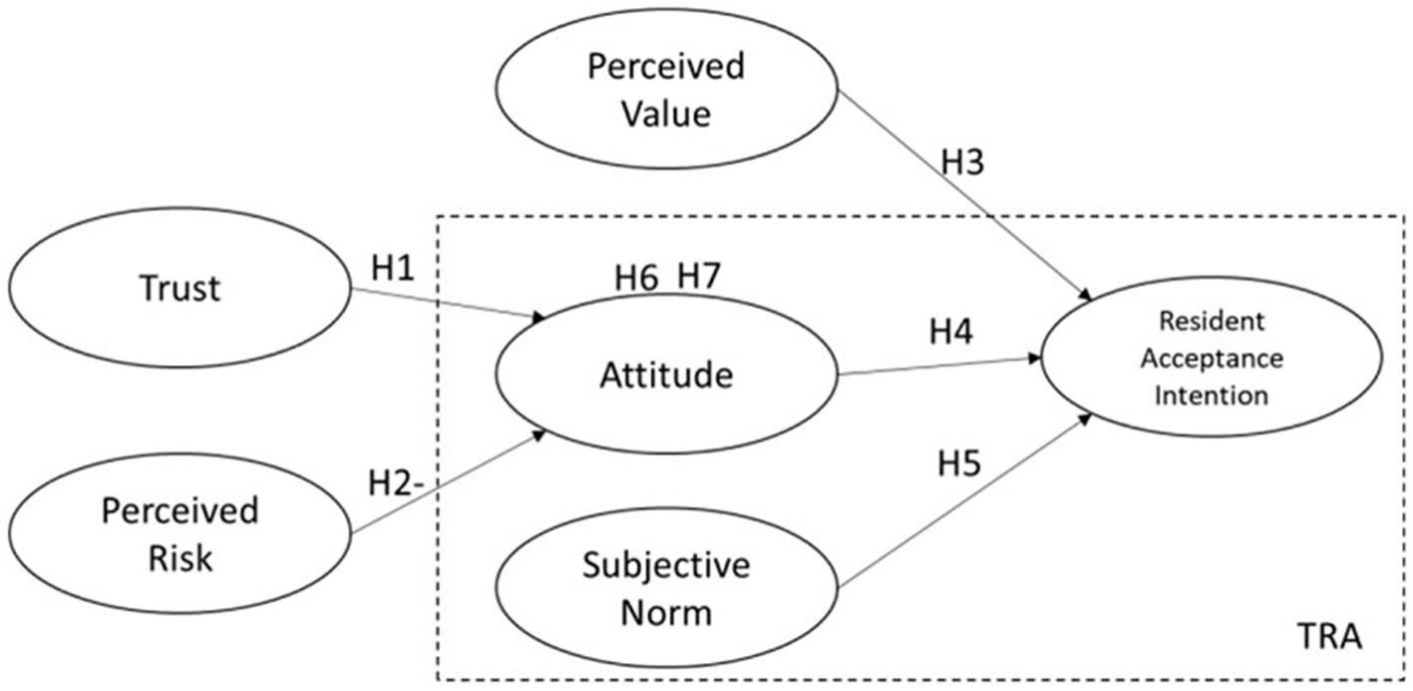
The theoretical model.

This study proposes a research framework as shown in Figure 1.

### Research design

#### Research subjects and data collection

This study applied a quantitative method using a questionnaire to collect data that is aimed at exploring the intention of residents to accept tourists after the COVID-19 pandemic. The target research group was residents in Hainan Province and the sampling design was based on the intentional sampling method. The questionnaire was distributed and filled out through paper and electronic questionnaires from 15 July to 10 November 2021, in the east, south, and north of Hainan Province. Data were collected from international companies, domestic enterprises and institutions, community groups, religious groups, civic associations, social media groups, and individuals. A total of 447 valid questionnaires were obtained. Based on the sample size requirement formula, a sample size of at least *N* = 384 for the overall population of Hainan Province was needed to meet a statistical confidence level of 95% confidence interval as suggested by The Survey System website (https://www.surveysystem.com/sscalc.htm).

#### Description of research variables

The questionnaire was divided into two major parts. The first part on basic personal information had six categories including gender, marital status, place of residence, monthly income, occupation, and education level. The second part on perceived risk and trust and acceptance intention by residents toward tourists after the COVID-19 pandemic had five variables of trust, perceived risk, perceived value, attitude, and subjective norm, in addition to resident acceptance intention.

#### Questionnaire design for the measurement of latent variables

The question measures were designed according to a 7-point Likert scale, with “1” strongly disagree, “2” disagree, “3” somewhat disagree, “4” average, “5” somewhat agree, “6” agree, and “7” strongly agree. The higher the score, the higher the level of agreement of the respondents for the study variables. After the questionnaire design was completed, tourism industry specialists and academic experts on tourism reviewed and augmented the questionnaire's content. The questionnaire contained 6 variables, 29 items, and literature sources as shown in Appendix.

##### Variable measures of acceptance intention

This study adapted the construct of acceptance intention from Fichten et al. (2016). The theory of planned behavior predicts the graduation intentions of Canadian and Israeli postsecondary students with and without learning. The original questions for the intention section were “I intend to complete my program of studies,” “I will try to complete my program of studies,” “I expect to complete my program of studies,” “I am determined to complete my program of studies,” and “All things considered, it is possible that I might not complete my program of study.” Corrections were made by translation, and a total of five questions were adopted from this study.

##### Variable measures of perceived value

This study referred to Liu et al. (2010) for questions on perceived value. Sample questions from the value of mobile phone services section include: “The service of the cell phone is good value for money,” “The service of mobile phone is a good buy,” “The price of mobile phone service is economical,” and “The service of the mobile phone is worthwhile.” The questions were revised and four questions were finalized.

##### Variable measures of perceived risk

The construct of perceived risk was adapted from Chen's (2012) study. The original intention of Chen (2012) was to examine the factors affecting the intention to continue using mobile banking. The original questions for the perceived risk section had sentences such as “Using m-banking to pay my bills would be risky,” “M-banking is dangerous to use,” “Using m-banking would add great uncertainty to my bill paying,” “Using banking exposes you to an overall risk,” and “On the whole, considering all sorts of factors combined, it is very risky if I sign up for and use m-banking.” A total of five questions were set.

##### Variable measures of perceived trust

The basis of our questions for perceived trust was formulated from Lin et al. (2010). This study investigated whether online team members can be trusted. The original questions for the trust were, “I consider our online team members as people who can be trusted,” “I consider our online team members as people who can be counted on to do what is right,” “I consider our online team members as people who can be counted on to get the job done right,” and “I consider our online team members as people who are always faithful.” The fifth question was designed by corrections and giving a summary by reference. A total of five questions were adapted.

##### Variable measures of attitudes

Questions for the construct of attitude were based on a 2017 study (Mehrad and Mohammadi, 2017). This study defined attitudes about the process of utilizing a mobile banking service. The original questions based on questions from the attitude section were, “Use mobile banking service is compatible with my lifestyle,” “Use banking services is compatible with most banking activities,” “Using mobile payment services is a wise idea,” and “Using mobile payment services is beneficial.” A total of five questions were set.

##### Variable measures of subjective norms

A 2018 study influenced the questions on subjective norms which explored behavioral intentions in recycling (Taufique and Vaithianathan, 2018). The questions from this study included, “Most of my friends think I should recycle household garbage,” “Most of my neighbors think I should use environmentally friendly household products,” “Most of my neighbors think I should recycle,” “Most of my co-workers think I should use environmentally friendly household products,” and “Most of my family members think I should use environmentally friendly products.” A total of five questions were designed.

#### Data analysis

Data analysis was conducted in three stages: descriptive analysis, measurement model verification, and structural equation modeling analysis. Descriptive analysis by SPSS contained two parts: the frequency distribution calculation of demographic data for a basic understanding of the sample set, and the mean value and standard deviation for each construct. A two-step analysis was then conducted to measure the model and structural model (Anderson and Gerbing, 1988), Reliability was confirmed through confirmatory factor analysis (CFA). This includes composite reliability which measures the internal consistency of each variable, convergent validity, and discriminant validity. In the third stage, a structural equation model (SEM) analysis was conducted to test the fit of the model and to check the hypotheses of the study structure. The structural equation model included factor analysis, path analysis, and mediation effect analysis by statistic software AMOS (SPSS Inc., Chicago, IL, USA).

### Study results

#### Descriptive analysis

##### Sample background data statistics

The basic data surveyed contained six items: gender, marital status, place of residence, monthly income, occupation, and education level. Among the respondents, *N* = 275 (61.52%) of them were women. A majority of the respondents were married, *N* = 264 (59.06%), and were residents of Haikou *N* = 273 (61.07%). Most of them had a monthly income of <5,000 RMB, *N* = 281 (62.86%). Many of them were occupied in jobs other than what was listed, *N* = 171 people (38.26%), and the majority had a bachelor's degree or a college degree, *N* = 272 (60.85%). The results are shown in Table 1.

**Table 1:** Frequency table.

**Variable**	**Value label**	**Frequency**	**Valid percent**	**CumPercen**
Gender	Male	172	38.48	38.48
	Female	275	61.52	100.00
Marital	Married	264	59.06	59.06
status	Unmarried	183	40.94	100.00
Place of	Haikou	273	61.07	61.07
residence	Sanya	57	12.75	73.83
	Wenchang	7	1.57	75.39
	Qionghai	9	2.01	77.40
	Wanning	15	3.36	80.76
	Others	86	19.24	100.00
Monthly	5,000 below RMB	281	62.86	62.86
income	5,001–10,000 RMB	103	23.04	85.91
	10,001–20,000RMB	35	7.83	93.74
	20,001 above RMB	28	6.26	100.00
Occupation	Staff of public institution/civil servant,	50	11.19	11.19
	State-owned enterprise employee,	23	5.15	16.33
	Foreign enterprise employee,	30	6.71	23.04
	Social enterprise	83	18.57	41.61
	Employee, businessman,	8	1.79	43.40
	Student	82	18.34	61.74
	Others	171	38.26	100.00
Education	High school and below	133	29.75	29.75
Level	Bachelor's degree or a college degree	272	60.85	90.60
	Postgraduate and above	42	9.40	100.00

##### Statistical analysis of question items

Table 2 displays the total number of valid questionnaires which was *N* = 447. The minimum value in the questionnaires was 1 and the maximum value was 7, and all values were within the range of 1–7, indicating no construction errors in the variables. The mean value was between 4.15 and 5.28, which met the criteria of no value being >6 or <2, which indicated all question items had discriminatory power. The analysis of the skewness and kurtosis for each question showed the range of skewness was −1.02−0.21 and the kurtosis values ranged from −0.91–0.54, which met the criteria of the absolute value of skewness of <2 and an absolute value of kurtosis <7 (Kline, 2005). This indicated that the study data conformed to normal distribution. The maximum mean value of PVQ1 was 5.28 and the minimum mean value of PRQ5 was 4.15, which meant that the respondents agree most with PVQ1 and less with PRQ5 (Table 2). The standard deviation ranged from 1.46 to 1.8, showing the degree of disagreement among the respondents was consistent for each topic.

**Table 2:** Overview of responses.

**Construct**	**Item**	**Mean**	**Std Dev**	**Kurtosis**	**Skewness**
Resident	INTQ1	4.75	1.80	−0.73	−0.57
acceptance	INTQ2	4.95	1.65	−0.29	−0.72
intention	INTQ3	4.72	1.75	−0.68	−0.49
	INTQ4	4.85	1.71	−0.57	−0.60
	INTQ5	4.90	1.76	−0.51	−0.69
Perceived value	PVQ1	5.28	1.53	0.47	−1.02
	PVQ2	5.03	1.57	−0.05	−0.80
	PVQ3	5.19	1.56	0.36	−0.95
	PVQ4	5.24	1.46	0.54	−0.94
Perceived risk	PRQ1	5.14	1.71	0.13	−1.02
	PRQ2	4.36	1.65	−0.61	−0.36
	PRQ3	4.71	1.70	−0.42	−0.66
	PRQ4	4.57	1.69	−0.51	−0.61
	PRQ5	4.15	1.75	−0.91	−0.21
Trust	TRU1	4.70	1.52	−0.29	−0.52
	TRU2	4.72	1.54	−0.35	−0.53
	TRU3	4.60	1.55	−0.40	−0.47
	TRU4	4.57	1.53	−0.45	−0.34
	TRU5	4.59	1.51	−0.34	−0.42
Attitude	ATT1	4.98	1.49	−0.05	−0.74
	ATT2	4.80	1.53	−0.29	−0.61
	ATT3	5.02	1.52	0.00	−0.75
	ATT4	5.02	1.51	0.04	−0.75
	ATT5	5.09	1.50	0.06	−0.77
Subjective norms	SN1	4.91	1.49	−0.12	−0.66
	SN2	4.81	1.49	−0.26	−0.54
	SN3	4.91	1.51	−0.15	−0.68
	SN4	4.81	1.54	−0.27	−0.61
	SN5	4.78	1.53	−0.34	−0.52

Std Dev, Standard deviation.

#### Reliability and validity analysis

According to Anderson and Gerbing (1988), a complete SEM evaluation consists of the evaluation of the measurement model and the evaluation of the structural model. Only when the measurement model passes the goodness-of-fit test can a complete SEM model analysis be performed. CFA is equivalent to the estimation of the measurement model in the SEM. In this study, the CFA measurement model was evaluated and modified according to the two-stage model (Kline, 2011).

The measurement model was estimated using an approximate estimation method. The estimated parameters included standardized factor loading, square multiple correlations (SMC), composite reliability, and average variance extracted (AVE) (Table 3). Among them, standardized factor loadings >0.60 are acceptable and ideally should be >0.70 (Chin, 1998). Few scholars have suggested that standardized factor loading questions below 0.45 indicate measurement error (Hair et al., 1998). In other words, the question is too broad and should be removed (Hooper et al., 2008). Other scholars have concluded that the standardized factor loading for each indicator variable should be >0.50, while composite reliability should be >0.60, and AVE should be higher than 0.50 (Fornell and Larcker, 1981; Nunnally and Bernstein, 1994). These parameters support that the measurement model will have good convergent validity.

**Table 3:** Analysis of measurement pattern results.

**Construct**	**Item**	**Significance of estimated parameters**				**Item reliability**		**Construct reliability**	**Convergence validity**
		**Unstd**.	**S.E**.	**Unstd./S.E**.	***p*-value**	**Std**.	**SMC**	**CR**	**AVE**
Resident acceptance intention	INTQ1	1.000				0.921	0.848	0.965	0.847
	INTQ2	0.914	0.027	33.611	0.000	0.914	0.835		
	INTQ3	0.978	0.028	34.456	0.000	0.920	0.846		
	INTQ4	0.957	0.028	33.756	0.000	0.916	0.839		
	INTQ5	0.991	0.028	35.647	0.000	0.931	0.867		
Perceived value	PVQ1	1.000				0.925	0.856	0.955	0.841
	PVQ2	1.008	0.030	33.669	0.000	0.912	0.832		
	PVQ3	1.025	0.029	35.341	0.000	0.928	0.861		
	PVQ4	0.937	0.029	32.457	0.000	0.904	0.817		
Perceived risk	PRQ1	1.000				0.758	0.575	0.935	0.744
	PRQ2	1.127	0.056	20.312	0.000	0.888	0.789		
	PRQ3	1.199	0.056	21.280	0.000	0.918	0.843		
	PRQ4	1.205	0.057	21.218	0.000	0.923	0.852		
	PRQ5	1.100	0.061	18.081	0.000	0.813	0.661		
Trust	TRU1	1.000				0.917	0.841	0.965	0.847
	TRU2	1.030	0.029	35.429	0.000	0.932	0.869		
	TRU3	1.029	0.031	33.715	0.000	0.921	0.848		
	TRU4	1.003	0.031	32.469	0.000	0.909	0.826		
	TRU5	1.010	0.030	34.071	0.000	0.922	0.850		
Attitude	ATT1	1.000				0.873	0.762	0.955	0.810
	ATT2	1.000				0.857	0.734		
	ATT3	1.073	0.037	28.973	0.000	0.919	0.845		
	ATT4	1.071	0.037	29.181	0.000	0.921	0.848		
	ATT5	1.077	0.036	29.670	0.000	0.929	0.863		
Subjective norms	SN1	1.000				0.940	0.884	0.973	0.878
	SN2	0.997	0.025	40.511	0.000	0.941	0.885		
	SN3	1.002	0.026	38.549	0.000	0.929	0.863		
	SN4	1.026	0.026	40.159	0.000	0.939	0.882		
	SN5	1.014	0.026	39.374	0.000	0.935	0.874		

Unstd., Unstandardized factor loadings; Std, Standardized factor loadings; SMC, Square Multiple Correlations.

CR: Composite Reliability; AVE: Average Variance Extracted.

As shown in Table 3, the standardized factor loadings ranged from 0.758 to 0.941, which represents that each item had topic reliability. Composite reliability (CR) was used as a reliability indicator for the constructs. Because CR values are suitable for data analysis of structural equation models, their role was similar to the function of Cronbach's alpha. The composite reliability for each construct ranged from 0.935 to 0.973. Previous studies have suggested that composite reliability should be >0.7, thus, all constructs met the criteria for good internal consistency. The AVE ranges were 0.744–0.878. Because all AVE ranges were above 0.5, all show good convergent validity for each construct (Fornell and Larcker, 1981; Hair et al., 1998).

Fornell and Larcker (1981) also suggested that discriminant validity should consider the relationship between convergent validity and construct correlation. Therefore, they suggested the square root of AVE for the construct should be greater than the correlation coefficient between the constructs. This would ascertain that the model in this study had discriminant validity. The root means square of the AVE for each construct on the diagonal is greater than the off-diagonal correlation coefficient (Table 4). Therefore, each construct in this study has good discriminant validity.

**Table 4:** Discriminant validity of measurement models.

	**Resident acceptance intention**	**Perceived value**	**Perceived risk**	**Trust**	**Attitude**	**Subjective norms**
Acceptance intention	**0.92**					
Perceived value	0.751	**0.917**				
Perceived risk	−0.198	−0.254	**0.863**			
Trust	0.706	0.760	−0.193	**0.92**		
Attitude	0.639	0.615	−0.227	0.799	**0.9**	
Subjective Norms	0.730	0.744	−0.168	0.797	0.638	**0.937**

The items on the diagonal in bold represent the square roots of the AVE; off-diagonal elements are the correlation estimates.

#### Structural equation modeling analysis

##### Structural model analysis

Structural model analysis was performed using the great likelihood estimation method. The analysis results include model fit, significance test of the research hypotheses, and explained variance (R^2^). The research hypothesis of SEM is sample covariance matrix = model covariance matrix. However, SEM is a large sample analysis method, so the *p*-value can easily be <0.05, which will often wrongly reject the hypothesis and conclude that the model is deficient. Schumacker and Lomax (2010) and Kline (2011) concluded the degree of model fit should not be determined by the *p*-value, but rather report a variety of different goodness-of-fit indicators to determine whether the model is good. Jackson et al. (2009), applied fit metrics to 194 international academic journals as a blueprint for applying model fit analysis. The results of this study reported the nine most widely used fit metrics: list the metrics here.

In principle, the lower the χ^2^ value the better, however, since χ^2^ is very sensitive to sample size, this study use χ2/df to reduce sensitivity and assist in the assessment. Ideally, this value should be <3. Hu and Bentler (1999) suggested each fit should be assessed independently and a more rigorous model fit should be used to control type I errors simultaneously. This includes Standardized RMR < 0.08 and CFI > 0.90 or RMSEA < 0.08. The model fit meets the criteria (Table 5), therefore, suggesting the model has a good fit.

**Table 5:** Model fit criteria and the test results.

**Model fit**	**Criteria**	**Model fit of research model**
MLχ^2^	The small the better	1,093.236
DF	The large the better	366.000
Normed Chi-sqr (χ^2^/DF)	1 <χ^2^/DF <3	2.987
RMSEA	<0.08	0.067
SRMR	<0.08	0.061
TLI (NNFI)	>0.9	0.953
CFI	>0.9	0.957
GFI	>0.9	0.937
AGFI	>0.9	0.930

RMSEA, Root mean square error of approximation; SRMR, Standardized root mean square residual; TLI (NNFI), Tucker-Lewis Coefficient (Non-normed Fit Index); CFI, Comparative Fit Index; GFI, Goodness of Fit Index; AGFI, Adjusted Goodness of Fit Index.

#### Path analysis

**Figure 2:**
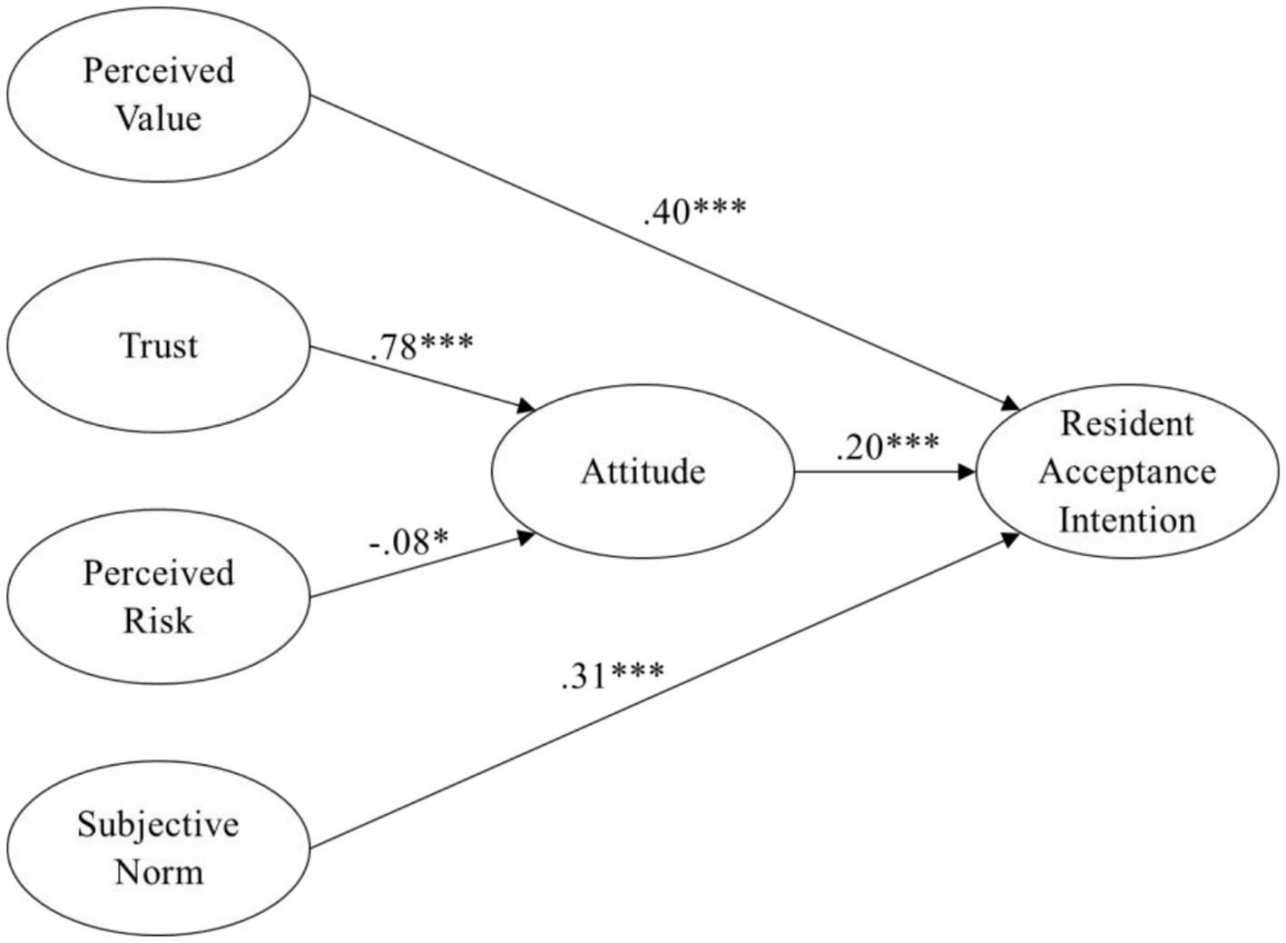
Statistical model with parameter estimates. *p < 0.05, ***p < 0.001.

In this research model (as shown in Table 6), Perceived value (PVQ) (b = 0.458, *p* < 0.001), attitude (ATT) (b = 0.240, *p* < 0.001), and subjective norm (SN) (b = 0.348, *p* < 0.001) significantly affect residents' acceptance intention (INTQ). Perceived risk (PRQ) (b = −0.078, *p* = 0.020) and trust (TRU) (b = 0.755, *p* < 0.001) significantly influence attitude (ATT). The explanatory power of perceived value (PVQ), attitude (ATT), and subjective norm (SN) in explaining was 65.1%. The explanatory power of perceived risk (PRQ) and trust (TRU) for explaining attitude (ATT) was 64.4%. Therefore, all hypotheses are established. Figure 2 represents the proposed conceptual model.

**Table 6:** Regression coefficient.

**DV**	**IV**	**Unstd**	**S.E**.	**Unstd./S.E**.	***p*-value**	**Std**.	***R* ^2^**
INTQ	PVQ	0.458	0.062	7.393	0.000	0.402	0.651
	ATT	0.240	0.067	3.605	0.000	0.197	
	SN	0.348	0.061	5.705	0.000	0.305	
ATT	PRQ	−0.078	0.033	−2.320	0.020	−0.076	0.644
	TRU	0.755	0.039	19.455	0.000	0.784	

TRU, Trust; PRQ, Perceived Risk; PVQ, Perceived Value; ATT, Attitude; SN, Subjective Norm; INTQ, Resident Acceptance Intention.

#### Analysis of mediation effect

The statistical confidence interval for the indirect effect was generated using bootstrapping, using resampling regression. Table 7 shows the indirect effect of perceived risk → resident acceptance intention is *p* ≥ 0.05 and the bias-corrected contains 0 [-0.074 to 0]. This means the indirect effect is not valid. In total, regarding the indirect effect of trust → resident acceptance intention, the confidence interval does not contain 0 [0.005–0.426], indicating that the indirect and mediating effect is maintained.

**Table 7:** Analysis of indirect effects of mediation effect.

**Effect**	**Point estimate**	**Product of coefficients**			**Bootstrap 1000 times**	
					**Bias-corrected 95%**	
		**S.E**.	**Z-Value**	***p*-value**	**Lower bound**	**Upper bound**
**Indirect effect**	
PRQ → ATT → INTQ	−0.019	0.017	−1.089	0.276	−0.074	0.000
TRU → ATT → INTQ	0.181	0.111	1.630	0.103	0.005	0.426

TRU, Trust; PRQ, Perceived Risk; PVQ, Perceived Value; ATT, Attitude; SN, Subjective Norm; INTQ, Resident Acceptance Intention.

## Discussion and conclusions with implications

This study explored the main variables influencing Hainan residents' acceptance intention regarding tourists after the COVID-19 pandemic, based on the theory of reasoned action, and combining the factors of “perceived value,” “trust,” and “perceived risk” with “attitude” as mediating variables. A research model and related hypotheses were proposed. After collecting data through questionnaires, SEM was used to test the model and verify the hypotheses.

### Theoretical contributions

The main aim of this study was to assess the acceptance intention of residents of Hainan to accept tourists in the context of the COVID-19 pandemic and to explore the influence of Hainan residents' perceived value on their acceptance intention, in addition to considering the influence of perceived risk and trust factors.

This study endeavors to fill the research gap on this subject by providing essential and empirical insights into the role of factors influencing tourist acceptance intentions post-COVID-19 era. First, trust affects the attitude of Hainan residents toward tourists. Trust as an independent variable is the most critical variable for tourists' acceptance in this study. This variable has a significantly positive effect on residents' intention to accept, with a standardized regression coefficient of 0.784. This aligns with Sadiq et al. (2021), who stated that one of the important variables in online travel purchases is “trust” and trust strongly influences consumers' attitudes toward online purchases. Most research scholars have explored the relationship between trust and intention from the consumer's perspective or the tourist's perspective on the product service and the physical product. This research explores the acceptance of tourists' wishes from the perspective of residents. The high trust level of residents directly reflects attitudes toward tourists. When residents have a friendly attitude to receiving tourists, naturally tourists will have a very pleasant experience.

Second, perceived risk affects the attitude of Hainan residents, and although it presents a significant negative effect, the effect is small. The findings of this study are similar to those of (Andrews et al., 2014), in that the findings show that perceived risk has a significantly negative effect on attitudes. As Polat et al. (2021) confirms, the variable with the greatest impact on explaining air travel intentions under the COVID-19 pandemic is perceived risk. To sum up, this study appears consistent with previous findings. The findings of this study also indicate Hainan residents are sympathetic and kind. This is a very important trait for a tourist city to have so guests can have a welcoming experience. Although the perceived risk of tourists is high, its impact coefficient is very small with a standardized regression coefficient of −0.076.

China's comprehensive protection against the pandemic, especially the government's implementation of a series of effective and rapid measures, is another important factor. For example, China's national real-name health code system network platform was rapidly established. Not only was a national platform on health code launched, but each local government also launched a provincial regional health code simultaneously and combined the two for integrated supervision. At the same time, personal information is required to be updated synchronously and the records inside the system include individual vaccination time details, nucleic acid antibody information, departure health information, travel track records, and vaccine prevention records. Effective and fast vaccination measures are believed to be the reason why Hainan residents show low rejection, no panic, and acceptance intention toward visitors. Thus, the importance of public media propaganda information mechanisms, timely updates to the masses, and external announcements of the latest COVID-19 pandemic case tracking and reporting reassured the public, easing the economic hardship period and fear of the COVID-19 pandemic. These are potential reasons why Hainan residents' intention to accept tourists is less affected by perceived risk.

Hainan residents have a significant positive influence on the perceived value acceptance intention of tourists. This is similar to the conclusion of previous studies which validate the positive effect of perceived value on air travel intentions (Polat et al., 2021). Um and Yoon (2021) identified the perceived value perception of tourism upscaling also had a significant impact on the willingness to conserve, indicating the importance of increasing the perceived value of tourist areas. Thus, with the pursuit of economic value and actual perceived value, Hainan residents typically accept tourists. Effective economic repair is very important for residents' daily economic livelihood and perceived value is one of the main considerations, especially for Hainan's geographical location and the Chinese government's tourism market orientation at the national level.

Based on the theory of reasoned action, it is clear that Hainan residents' acceptance intention toward tourists has been directly influenced by subjective norms. This study verifies the influence of the relationship between attitude, subjective norms, and intention. The conclusion indicates Hainan residents are more compliant with local resident norms and actively cooperate with a series of national and governmental legal measures for the COVID-19 pandemic. Residents are also very dependent on local governmental legal regulations for tourist acceptance intentions, including at the workplace, local community, and influence of surrounding family and friends.

### Substantive contributions

The results of this study can effectively assist various stakeholders including local governments, the tourism industry, the hotel industry, and the restaurant industry. First, this study found that the attitude and acceptance of Hainan residents toward tourists are high. Therefore, the local government has been very successful in promoting and educating residents about the attitudes and acceptance of tourists. The paper recommended continuing to promote and educate tourists in a friendly manner. Furthermore, perceived value has a significantly positive effect on local residents' acceptance of tourists. Therefore, it is recommended for the local government, tourism industry, hotel industry, and catering industry that these stakeholders should adopt a series of reasonable and effective strategies, product branding effect, and destination attachment to persuade tourists to choose higher levels of products or services. Standardization and improvement of tourism support facilities provide tourists with friendlier, and better quality tourism services in the local market. Second, based on the recommendations from the study, the focus should be on the development of various types of services including experiential consumption, diversification of innovative tourism consumption products, effective targeting of personalized tourism products, dynamic market demand for tourism services, integrated marketing communications, multi-directional information placement to stimulate tourist consumption, and constantly achieve demand-oriented attraction of tourists. Finally, based on the comprehensive needs of residents and visitors, this paper provides rich and professional practical training services and enhances accessibility to residents.

Another major finding is that perceived risk has a direct negative influence on attitudes, but the influence is small. Hainan residents show a low perceived risk of rejection by visitors indicating the local government has done well to provide comprehensive protective measures, a series of security services, and a safe and secure system for the COVID-19 pandemic, allowing for low-risk perception in tourists. It is recommended that such benign precautionary measures and local safety and security services be maintained continuously. It is believed that such measures are the reason residents are at ease and willing to accept tourists.

### Research limitations and future developments

This study is mainly for Hainan residents, and only the core cities in Hainan Province were selected for sampling such as Haikou, Sanya, Wanning, and Wenchang. The regional selection does not cover the whole Hainan province. Results learned for data of the sampling target Haikou city is relatively high at 61.07% and there is unevenness in the data between cities. College and university-educated residents accounted for a total of 60.85%. The higher the education level, the more responses, and flexibility to different emergency scenarios came easier, and the higher the willingness to easily accommodate visitors in the context of the pandemic. This group also has a higher percentage of people who know how to effectively use the online real-name health code and how to use safety preparation in special situations such as the pandemic. The data report may not fully present the attitudes and intentions of other people with lower education. Furthermore, the concept of variables itself can be extended for the limited number of variables in the construct design. For example, perceived risks can be subcategorized and safety SOP norms can be explored as a separate column. In addition, this study focuses on current Hainan residents, and in the future, the methods and conclusions from this study could apply to other regions of China or other countries, especially tourist destinations. Expanding the understanding of the willingness of residents of various countries to accept tourists in the context of the COVID-19 pandemic is useful in the future.

## Data availability statement

The raw data supporting the conclusions of this article will be made available by the authors, without undue reservation.

## Ethics statement

Ethical review and approval was not required for the study on human participants in accordance with the local legislation and institutional requirements. Written informed consent from the patients/participants or patients/participants legal guardian/next of kin was not required to participate in this study in accordance with the national legislation and the institutional requirements.

## Author contributions

HZ devised the project, the main conceptual ideas, proof outline and all of the technical details for data analysis. JI worked out writing suggestion and guideline, with help from AM. All authors contributed to the article and approved the submitted version.

## Conflict of interest

The authors declare that the research was conducted in the absence of any commercial or financial relationships that could be construed as a potential conflict of interest.

## Publisher's note

All claims expressed in this article are solely those of the authors and do not necessarily represent those of their affiliated organizations, or those of the publisher, the editors and the reviewers. Any product that may be evaluated in this article, or claim that may be made by its manufacturer, is not guaranteed or endorsed by the publisher.

## Appendix

**Appendix:** Summary of construct measures.

**Construct**	**Items**	**Item content**	**literature sources**
Resident	INTQ1	1. I intend to accept the tourists.	Fichten et al., 2016
Acceptance Intention	INTQ2	2. I will try to accept the tourists.	
	INTQ3	3. I expect to accept the tourists.	
	INTQ4	4. I am determined to accept the tourists.	
	INTQ5	5. All things considered I completely accept tourists.	
Perceived Value	PVQ1	1. The tourists are good value for money.	Liu et al., 2010
	PVQ2	2. The tourists are a positively benefit local residents.	
	PVQ3	3. The tourists are economical.	
	PVQ4	4. The tourists are worthwhile.	
Perceived Risk	PRQ1	1. The tourists during a COVID-19 pandemic would be risky to local residents.	Chen, 2012
	PRQ2	2. The arrival of tourists is dangerous to local residents.	
	PRQ3	3. Tourists adds great uncertainty for local residents.	
	PRQ4	4. The tourists expose an overall risk to local residents.	
	PRQ5	5. All things considered, tourists are very risky to local residents.	
Trust	TRU1	1. I consider tourists as people who can be trusted.	Lin et al., 2010
	TRU2	2. I consider tourists as people who can be counted on to do what is right.	
	TRU3	3. I consider tourists as people who can be counted on to get the job done right.	
	TRU4	4. I consider tourists as people who are always faithful.	
	TRU5	5. Overall, I think that tourists are trustworthy to local residents.	
Attitude	ATTI1	1. Having tourists is compatible with my lifestyle.	Mehrad and Mohammadi, 2017
	ATTI2	2. Tourists is compatible with most local resident activities	
	ATTI3	3. Opening services to tourists is a wise idea	
	ATTI4	4. Tourists are beneficial to local residents.	
	ATTI5	5. In general, I welcome tourists.	
Subjective Norm	SN1	1. Most of my friends think I should accept tourists.	Taufique and Vaithianathan, 2018
	SN2	2. Most of my neighbors think I should accept tourists.	
	SN3	3. Most of my co-workers think I should accept tourists.	
	SN4	4. Most of my family members think I should accept tourists.	
	SN5	5. Most of my local community members think I should accept tourists.	

Data source: compiled by this study.
